# Climate drivers of the Amazon forest greening

**DOI:** 10.1371/journal.pone.0180932

**Published:** 2017-07-14

**Authors:** Fabien Hubert Wagner, Bruno Hérault, Vivien Rossi, Thomas Hilker, Eduardo Eiji Maeda, Alber Sanchez, Alexei I. Lyapustin, Lênio Soares Galvão, Yujie Wang, Luiz E. O. C. Aragão

**Affiliations:** 1 Remote Sensing Division, National Institute for Space Research - INPE, São José dos Campos 12227-010, SP, Brazil; 2 CIRAD, UMR Ecologie des Forêts de Guyane, Kourou 97379, France; 3 UR B&SEF Biens et services des écosystèmes forestiers tropicaux, CIRAD, Yaoundé BP 2572, Cameroon; 4 Department of Geography and Environment, University of Southampton, Southampton SO17 1BJ, United Kingdom; 5 Department of Environmental Sciences, University of Helsinki, Helsinki, FI-00014, Finland; 6 Earth System Science Center, National Institute for Space Research - INPE, São José dos Campos 12227-010, SP, Brazil; 7 Goddard Space Flight Center, NASA, Greenbelt, MD 20771, United States of America; 8 College of Life and Environmental Sciences, University of Exeter, Exeter, EX4 4RJ, United Kingdom; Montana State University Bozeman, UNITED STATES

## Abstract

Our limited understanding of the climate controls on tropical forest seasonality is one of the biggest sources of uncertainty in modeling climate change impacts on terrestrial ecosystems. Combining leaf production, litterfall and climate observations from satellite and ground data in the Amazon forest, we show that seasonal variation in leaf production is largely triggered by climate signals, specifically, insolation increase (70.4% of the total area) and precipitation increase (29.6%). Increase of insolation drives leaf growth in the absence of water limitation. For these non-water-limited forests, the simultaneous leaf flush occurs in a sufficient proportion of the trees to be observed from space. While tropical cycles are generally defined in terms of dry or wet season, we show that for a large part of Amazonia the increase in insolation triggers the visible progress of leaf growth, just like during spring in temperate forests. The dependence of leaf growth initiation on climate seasonality may result in a higher sensitivity of these ecosystems to changes in climate than previously thought.

## Introduction

The Amazonian forests account for 14% of the global net primary production (NPP) and are a major component (66%) of the inter-annual variation in global NPP [1]. While large seasonal swings in leaf area have been reported at least in parts of the Amazon basin [2–4], the environmental controls that trigger the synchronous development of new leaves are not well understood [5–7]. As a result, current earth system models inadequately represent the dynamics of leaf development, despite its major role for photosynthesis of tropical vegetation [8]. In equatorial forests, leaf flushing correlates with increased light availability and photosynthetically active radiation during the dry season [4, 9], and is theoretically driven by a change in daily insolation [10]. However, water availability constrains leaf phenology in southern Amazonia and most of the Congo basin, impeding the maintenance of the evergreen state during the dry season [11]. The climate thresholds controlling the phenological responses of vegetation remain unclear [12], as well as the sensitivities of these responses to future climate changes [13].

The Enhanced Vegetation Index (EVI), from the Moderate Resolution Imaging Spectroradiometer (MODIS), improves the sensitivity of the vegetation signal in high biomass regions and it strongly correlates with chlorophyll content and photosynthetic activity [5, 14]. An unexpected result from this index was the Amazon forest “greening” in the dry season [5]. The evidence provided by the MODIS sensor raised a debate not on the fact of the dry-season greening of the forest, but on deciphering if this greening is more intense during drought years or not [6, 15]. Later, it has been suggested that the observed greening during the dry season in the Amazon could be attributed to artefact of sun-sensor geometry [16, 17]. However, a new version of EVI supports the dry season greening in the Amazon [4, 18]. This EVI is corrected for the sun-sensor artefact from the multi-angle implementation of atmospheric correction algorithm (MAIAC) [19]. Furthermore, a recent analysis of seasonal canopy leaf area index (LAI) changes estimated from independent lidar-based satellite observations over the Amazon forest fully supports the existence of the greening [20].

While the biophysical interpretation of EVI increase remains to be fully assessed [21], recent accumulation of evidences in the Amazon converge to a positive correlation of EVI increase and leaf flushing. This have been observed with different types of field data such as percentage of trees with new leaves [18, 22, 23], green crown fraction measured above the canopy with camera [8], indirect estimation of leaf flush with litterfall [24, 25], leaf spectral reflectance measurements [26] and remote-sensed changes of lidar-derived canopy leaf area index (LAI) [20].

The observations of leaf-flushing during the dry season challenge our vision of droughts in tropical forests. The droughts may have negative impact on the forest structure and dynamics [27], such as increase in mortality [28, 29] or reduction of tree radial growth [25]. Furthermore, tropical trees show at some extent adaptation to drought with leaf water potential at wilting point ranging from -1.4 to -3.2 MPa [30]. But, in case of water stress, a common behavior of the trees is to shed the leaves, as observed in dry tropical forest [31]. This indicates that leaf-flushing in the heart of the dry season necessarily implies a sufficient supply of water. Above ∼2000 mm of annual precipitation, global tropical forests maintain evergreen state and present the increase of the photosynthetic capacity—the greening—during the driest season [11]. These forests, whose canopies’ processes are observed using remote sensing, are not limited by water; they satisfy their water demands during the dry season using the supply of redistributed subsurface water of the wet season [11]. Despite the leaf phenology of Amazonian forests appears driven by sun-related variables and shows greening during dry seasons, an analysis of the potential drivers in a continuous spatial framework is still missing [4, 5, 9, 20].

To investigate the Amazon seasonality, we model the period of increase in leaf production, proxied by the main period of increasing remotely sensed Enhanced Vegetation Index (EVI) [14, 32], with the seasonal increase of the potential climate drivers, *i.e.* precipitation and insolation (proxied by maximal temperature, S1 Fig). We assume that EVI “greening” relates to seasonal flush of new leaves based on recent evidences obtained in Amazonia [8, 20, 22, 23, 26]. EVI values were computed from the multi-angle implementation of atmospheric correction algorithm (MAIAC) [19]. We used EVI normalized to a nadir view angle and 45°sun zenith angle to avoid artifacts from changing sun-sensor geometry over time.

## Materials and methods

### Satellite greenness from MODIS enhanced vegetation index (MAIAC EVI)

We used enhanced vegetation index (EVI) imagery obtained by the Moderate Resolution Imaging Spectroradiometer (MODIS) sensor, on board the Terra and Aqua satellites (EOS AM, NASA) [33]. Data were processed with multiangle implementation of atmospheric correction algorithm (MAIAC) [19]. Data were obtained for 12 MODIS tiles (h10v08 to h13v10, spanning 10°N to 20°S in latitude and 80°W to 40°W in longitude) from NASA’s Level 1 and Atmosphere Archive and Distribution System (LAADS Web: ftp://ladsweb.nascom.nasa.gov/MAIAC). MAIAC observations are based on MODIS Collection 6 Level 1B (calibrated and geometrically corrected) observations, which removed major sensor calibration degradation effects present in earlier collections. We used observations from the Terra and Aqua satellites collected between 2000 and 2012 at 1-km spatial resolution. EVI data were corrected for bidirectional reflectance effects by normalizing all observations to a fixed sun sensor geometry (solar zenith angle of 45° and nadir view angle) [19]. Advanced cloud detection and aerosol-surface retrieval in MAIAC improves the accuracy of satellite-based surface reflectances over tropical vegetation 3 to 10 fold compared with the standard MODIS products [34].

### Climate measurements

Precipitation measurements were obtained from the Tropical Rainfall Measuring Mission (TRMM) 3B43V7 product, which provides monthly precipitation estimates at 0.25° spatial resolution, for the tropical and subtropical regions. We analyzed data from 2000 to 2013. Maximal temperature (*tmx*, °C) was obtained from monthly climate global dataset (CRU-TS3.21) available at 0.5° spatial resolution, from 1901–2012, produced by the Climate Research Unit (CRU) at the University of East Anglia [35]. Additionally, we used monthly incoming solar radiation at the surface (*rad*, W.m^−2^) covering the period 2000–2012 at 0.5° spatial resolution, estimated by the Clouds and the Earth’s Radiant Energy System (CERES) instruments onboard the NASA Terra and Aqua satellite [36]. In preliminary analysis, we compared maximal temperature from CRU and incoming radiation at the surface from CERES data. Maximal temperature from CRU was highly correlated with incoming radiation at the surface from CERES (Pearson’s r = 0.76, p-value < 0.0001, S1 Fig). Here, we used maximal temperature rather than incoming radiation because it has previously been shown to be a good predictor of EVI seasonality in tropical forests [25].

### Vegetation land mask and altitude datasets

EVI pixels covering areas with tree cover below 80% were excluded from the analysis. Forest masking was done using the Global forest cover loss 2000–2014 data set based on Landsat data at 30 m spatial resolution [37]. We used the vegetation map for Brazil [38] to analyze if the seasonal patterns of EVI were related to vegetation type. Additionally, to test the effect of the altitude on the quality of our EVI model, we used altitude from the Shuttle Radar Topography Mission (USGS/NASA SRTM data), resampled to 250 m spatial resolution for the entire globe (CGIAR-CSI, version 4.1) and filled to provide seamless continuous topography surfaces [39].

### Field measurements of litterfall productivity

Seasonal litterfall productivity measurements from two previously published studies were used to estimate leaf-fall productivity [25, 40] (Table 1). It has been previously demonstrated that the seasonal pattern of total litterfall (leaves, fruits, flowers and twigs) in this dataset is not different from seasonal pattern of leaf-fall alone (Pearson test, t = 42.7597, df = 218, p-value < 0.001) [25]. Furthermore, no relation between seasonal litterfall and soil types has been observed in this dataset [40]. Here, we used only data with monthly measurements from old-growth forests, as some sites have plots of both secondary and old-growth forests and flooded forests were excluded. Only the sites where the model of EVI increase had a R^2^ above 0.8 were selected. While these measurements are from the largest litterfall database ever produced for South America, some of the measurements have been made before the MODIS era.

**Table 1:** Description of the study sites for litterfall measurements, adapted from [25]. For each site, reference of the articles, country, full site name and geographical coordinates (longitude and latitude in decimal degrees) are reported. The next columns reports the type of measurements, only leaf fall (YES) or total litterfall (NO), the number of traps, the trap size, the total area sampled, the mean litterfall productivity in Mg.ha^−1^.year^−1^ and the duration.

reference	country	site	Latitude	Longitude	type	trap nb	trap size	tot size	Mean ± SE	duration
[25, 40]	Brazil	BDFFP Reserve	-2.500	-60.000	NO	18	1	18	6.59±0.675	1999/2002
[25, 41]	Brazil	Caracarai	1.476	-61.019	YES	75	0.25	18.75	5.36±0.19	2012/2013
[25, 40]	Brazil	Caxiuana	-1.785	-51.466	YES	25	0.25	6.25	6.17±0.738	2005/2006
[25, 40]	Brazil	Cuieiras Reserve Manaus	-2.567	-60.117	NO	15	0.5	7.5	8.03±0.564	1979/1982
[25, 40]	Brazil	Ducke	-2.952	-59.944	YES	10	0.25	2.5	3.97±0.197	1976/1977
[25, 40]	Brazil	Jari Para	-1.000	-52.000	YES	100	0.25	25	7.63±0.896	2004/2005
[25, 40]	Brazil	Manaus	-3.133	-59.867	NO	20	0.25	5	7.24±0.607	1997/1999
[25, 42, 43]	Brazil	Marajoara	-7.833	-50.267	NO	50	1	50	3.53±0.416	1998/2001
[25, 40]	Brazil	Rio Juruena	-10.417	-58.767	YES	16	1	16	5.21±1.514	2003/2004
[25, 40]	Brazil	Sinop	-11.412	-55.325	YES	20	1	20	5.27±1.116	2002/2003
[25, 44, 45]	Brazil	Tapajos km83	-3.017	-54.971	YES	30	1	30	5.54±0.533	2000/2003
[25, 40]	Colombia	Amacayacu	-3.717	-70.300	YES	25	0.5	12.5	6±0.31	2004/2006
[25, 40]	French Guiana	Nouragues	4.084	-52.680	YES	40	0.5	20	5.88±0.64	2001/2008
[24, 25, 46, 47]	French Guiana	Paracou	5.279	-52.924	YES	40	0.45	18	4.77±0.311	2003/2011
[25, 40]	French Guiana	Piste de Saint Elie	5.333	-53.033	YES	60	1	60	5.04±0.608	1978/1981
[25, 40, 48]	Peru	Tambopata	-12.835	-69.285	YES	25	0.25	6.25	7.16±0.607	2005/2006

### Data preparation

The spatial resolution of all data sets, including climate variables, tree cover, land mask and altitude, were resampled to 1 km, to match the resolution of MAIAC EVI. Inter-annual monthly mean values of EVI were filtered using the Fourier Transform (FT) to keep only the annual and bis-annual frequencies that compose the EVI signal (S1 Appendix). Maximal temperature and precipitation inter-annual monthly mean values were computed by pixel. For the analysis, we computed normalized EVI MAIAC (FT filtered), maximal temperature and precipitation by subtracting the mean and dividing by the standard deviation.

### Models

#### Individual pixel EVI model

For a pixel *i*, *i* = 1, …, *n*, normalized EVI (*EVIn*) was modeled using normalized precipitation (*pre*) and normalized maximal temperature (*tmx*) as:

$$\begin{array}{c} EVIn_{i,t}=\alpha_{0}^{i}+\alpha_{1}^{i}\times pre_{i,t}+\alpha_{2}^{i}\times tmx_{i,t-lag}+\varepsilon_{i,t} \end{array}$$ (1)

where $\alpha_{1}^{i}\geq 0$, $\alpha_{2}^{i}\geq 0$, $\varepsilon_{i,t}∼N\left(0,\sigma^{2}/\omega_{i,t}\right)$ and *t* = 1, …, *n* is a given month. Here, we assume that EVI increase is a proxy for the production of new leaves [8, 23, 26]. To focus our evaluation on the effects of leaf flushing rather than leaf aging, we limited the subsequent analysis to the increasing part of EVI, rather than the decreasing one, which is generally related to leaf aging (changes in morphological and biochemical leaf traits) and parasites [26, 32]. To account for this, the coefficients of the model $\alpha_{1}^{i}$ and $\alpha_{2}^{i}$ were set to have non-negative values. To give more importance to the fitting of the main increasing EVI period, we add an arbitrary weight, i.e. *ω*_*i*,*t*_ = 10 during a month *t* of the “main increase” and *ω*_*i*,*t*_ = 1 otherwise. The “main increase” was define as the longer period between a pit and a peak in the EVI time series (S1 Appendix). Finally, we minimized the weighted sum of squares of the model. This enables us to use all months to fit the model, including the EVI decreasing months, which resulted in a better model fit.

$$\begin{array}{ccc} residuals_{i,t} & = & EVIn_{i,t}-{\hat{EVIn}}_{i,t} \end{array}$$ (2)

$$\begin{array}{ccc} SSe_{i} & = & \sum_{t=1}^{12}\left(\omega_{i,t}\times residual_{i,t}^{2}\right) \end{array}$$ (3)

$$\begin{array}{ccc} SSt_{i} & = & \sum_{t=1}^{12}\left(\omega_{i,t}\times {\left(EVIn_{i,t}-\left(\sum_{t=1}^{12}w_{i,t}\times EVIn_{i,t}/12\right)\right)}^{2}\right) \end{array}$$ (4)

$$\begin{array}{ccc} R_{i}^{2} & = & 1-(SSe_{i}/SSt_{i}) \end{array}$$ (5)

For each pixel *i*, the coefficient of determination $R_{i}^{2}$ was computed with the weights *ω*_*t* = 1: 12,*i*_, that is, the residual sum of squares (*SSe*_*i*_, Eq 3) and the total sum of squares (*SSt*_*i*_, Eq 4) were weighted. *EVIn* is the observed normalized EVI and $\hat{EVIn}$ its prediction from the linear model.

The determination of the time lag between the increase of maximal temperature and EVI increase was made in two steps. First, we estimated the best EVI model for the whole area with a lag of 0, 1, 2 and 3 months. Second, we estimated the best model of EVI increase with the increase of maximal temperature at lag 0, 1, 2 and 3 months by pixels, by using only the pixels where precipitation and maximal temperature are correlated below 0.2 during the increase of EVI at all the lags. This selection discards artificial correlations between the climate variables. Then, we compared the results by pixels of the best model selected at the first step for the whole area (one month) and the lags obtained at the second step using the Bayesian information criterion [49]. Finally, we kept the best model for each pixel.

Monthly leaf fall estimation (from monthly litterfall productivity measurements) were normalized and smoothed using a spline with 4 degrees of freedom and compared to the normalized EVI (*EVIn*).

Finally, the quality of the fit of the EVI model was analyzed by comparing it against the mean annual precipitation, the mean annual maximal temperature and standard deviation, the mean percentage of valid observations per month in MAIAC data, the periodicity of the EVI signal, the altitude and the tree cover.

All analyses were performed using R [50]

## Results

**Fig 1:**
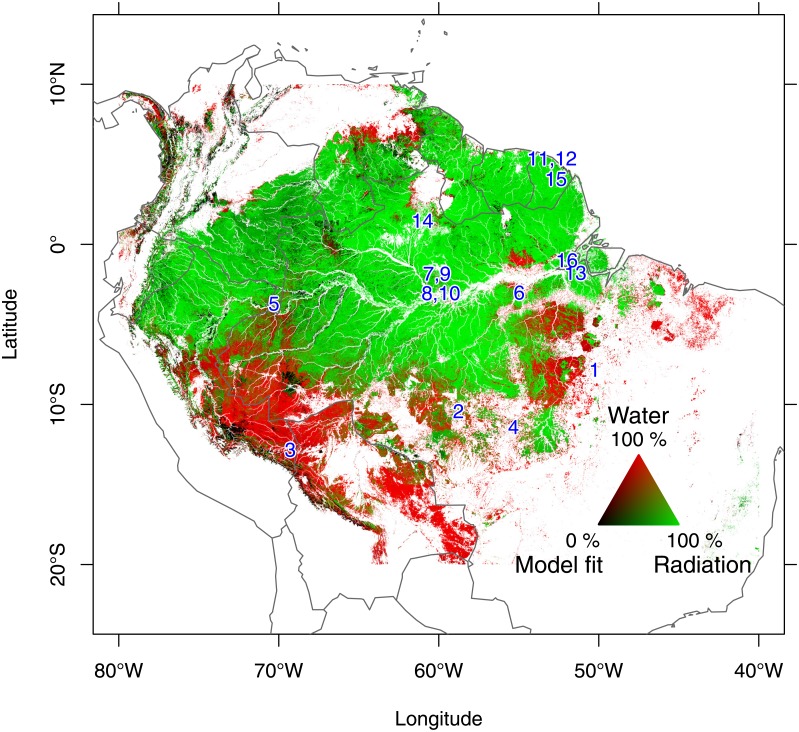
Spatial pattern of climate controls on leaf growing season initiation in South American tropical forests. Locations of the sites with monthly litterfall productivity measurements are indicated by blue numbers.

EVI greening across the Amazon forest can be explained by seasonal increase in insolation and precipitation (Fig 1 and S8 Fig). Seasonal changes in EVI were mainly related to insolation (70.4% of the total area) in the north-western, central and eastern part of Amazonia. EVI greening is associated to precipitation seasonality (29.6% of the area) in continuous areas above 5°N (North part) and below 5°S (South-West, South and South East part). 75.0% of the pixels where the greening is associated to insolation follows the increase in solar radiation with a mean lag of one month (S7 Fig). No time lag was observed in regions where greening is associated to an increase in precipitation. The modeled EVI with precipitation and maximal temperature is consistent with MAIAC EVI (S8 Fig). Inconsistent regions (R^2^<0.5, 6.2% of the forested area) have either uncommon forest structures, such as bamboo dominated forests, or constant cloud cover inducing high noise in the EVI signal (S3 Appendix, S10 Fig)

**Fig 2:**
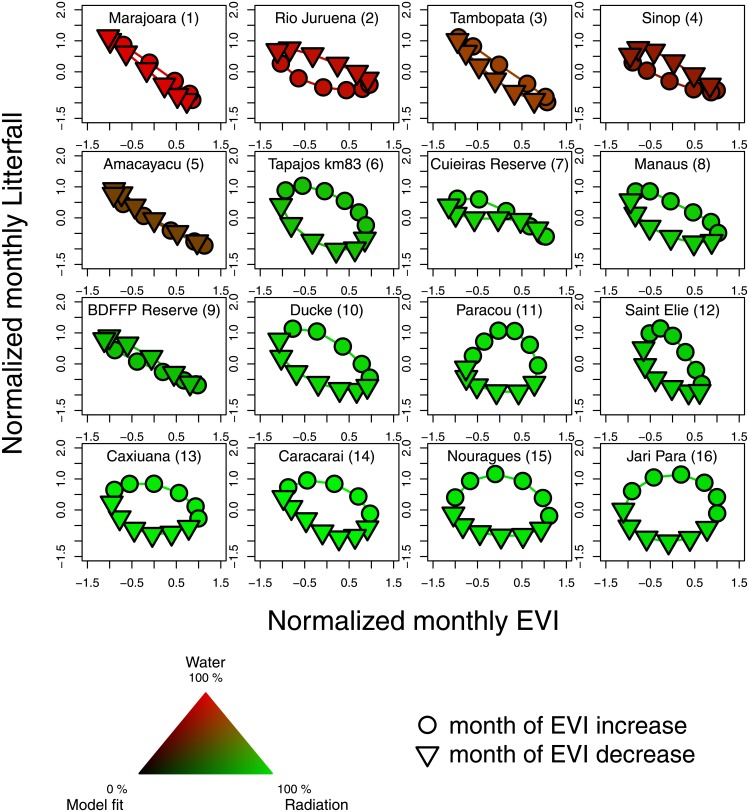
Seasonal leaf production (EVI increase) and associated litterfall productivity in 16 tropical forest sites.

The seasonal association of MAIAC EVI and litterfall (measured across 16 sites within the Amazon basin, Table 1), changes depending on the climate variable associated with EVI (Fig 2). In the sites where EVI is associated to precipitation, EVI has an negative linear relation with litterfall (site 1-5, Fig 2). In these sites, the browning of the vegetation occurs when leaf shedding is maximum. For most of the sites where EVI is associated to radiation a singular temporal pattern is observed. The highest production of litterfall occurs, not when EVI is maximum, but when EVI increase (site 6-16, Fig 2). All these sites present a peak of litterfall even if some of them produce litterfall in all the months of the year.

## Discussion

Despite the high diversity and different species composition of these forests, we show that EVI greening across the Amazon forest may be explained by climate seasonality (Fig 1). The predicted EVI from precipitation and insolation is highly consistent with the observed MAIAC EVI (S8 Fig), supporting the model’s biological assumptions: EVI increase is likely a proxy of leaf production triggered by climate drivers. Isolated patches where the results are not consistent (R^2^<0.5, 6.2% of the forested area) represent areas highly challenging for remote sensing; that is, uncommon forest structures, such as bamboo dominated forests, constant cloud cover resulting in high noise in EVI (S3 Appendix and S10 Fig); and may be micro-climate or local soil water retention characteristics. Comparing our modeled EVI to MAIAC EVI reveals limitations of the remotely sensed EVI that cannot be discovered with classic remote sensing analysis (such as classifications) which does not consider the biophysical nature of the signal. Additionally, it gives a biological interpretation of EVI derived from optical measurements of reflected electromagnetic radiation.

It was previously shown that the difference of mean wet and dry season EVI (Δ*EVI*_(*wet*−*dry*)_) is associated to water availability for the canopy processes in tropical forests, globally [11]. The authors found that Δ*EVI*_(*wet*−*dry*)_ is positive in forests with precipitation < 2000 mm.yr^−1^, while negative in region with no water limitations. Our results agree with this study. In water-limited regions, where precipitation is the main control of EVI seasonality, the lowest EVI is observed during the driest period. Additionally, here we show that for forests without water limitations (highest EVI during the dry periods), seasonal radiation increase is likely the climate control of EVI greening, as already observed for several sites in tropical forests [4, 5, 25, 51]. These results refute the general intuition found in the literature regarding the negative effect of dry seasons or seasonal droughts on tropical forest dynamics [27]; for example, seasonal or temporary droughts are known to increase the mortality of trees [28, 29] and to reduce tree growth [25]. However, we show that the dry season consequences on the canopy functioning is not always stress. This is confirmed by dry season observations of leaf flushing [8, 20, 22, 23, 31, 52, 53]; and photosynthesis increase from flux towers [9, 54] and satellite remote sensing [4, 5, 11, 18, 51]. This indicates that during the dry season, these forests might not be limited by water. From ours and previous results based on EVI [11], we further referred to water-limited and light-limited for the forest where EVI seasonality is associated to water or radiation, respectively.

In light-limited forests, EVI peaked during the period of increasing insolation, likely as a result of increased new leaf production [3, 5–7, 11, 22, 23, 51]. While not all the species have annual leaves production cycle, field observations of proportion of trees with news leaves in light-limited forest support this result [8, 18, 22]. Here, the greening follows the increase in solar radiation with a mean time lag of one month (75.0% of the light limited pixels, S7 Fig); which is consistent with dry season field and satellites observations of leaf flushing, which occurred several weeks after increase in insolation in tropical forests [9, 23, 51, 55, 56]. The bud break preceding synchronous greening of tropical forests are caused by an environmental signal perceived weeks before leaf emergence and this has been ignored by previous remote sensing studies [10, 57]. The time lag observed here may have three explanations. First, it could be caused by the sensitivity of trees to seasonal changes in climate. A gradual increase in insolation might be a climate signal more subtle to perceive than the rapid transition from dry to wet season, and could explain why leaf flush is not synchronized with the increase of insolation. Second, in the absence of a strong signal during the dry season, the observed time lag may also be related to the production of bud and subsequent bud break if the buds have not been produced on the trees during the rainy season and need time to develop following the increase of insolation. Third, the time lag could reflect yet-unknown endogenous driver of leaf production. For instance, we know that individuals of the same species can present differences in the timing of leaf flush [53] and that leaf renewal and/or net leaf abscission also occur during the entire year unrelated to climate variations [3, 4, 10, 25, 51]. The 1km spatial resolution of EVI MAIAC integrates the changes in the canopy of all the trees in this area. While it exists a variability between species and individuals, remarkably, the change of the canopy properties visible from space seems to indicate that in light-limited forests an important proportion of the trees flush their leaves during the dry season. The time lag may thus be pragmatically viewed as the mean response time between insolation increase and the flushing of new leaves.

In water-limited regions, greening is associated with an increase in precipitation. This is consistent with observations in dry tropical forests, where the timing of synchronous bud break of leafless trees varied from year to year with the first rains of the wet season, as irrigation caused bud break within a few days [58]. This also suggests that the leaf buds are already set up but stay dormant during the dry season. The relationship between seasonal changes in MAIAC EVI and litterfall (Fig 2) confirms two major assumptions about leaf fall seasonality in tropical forests. First, abscission due to the seasonal production of new leaves in response to increased light availability when water is not limited, and second, leaf shedding due to high evaporative demand in water limited environments [3, 8, 25, 40, 51, 59, 60].

In water-limited forests where the seasonal leaf fall responds to high evaporative demand [25], the leaf fall peaks when minimum EVI values are observed. The linear relation between EVI and leaf fall suggests that the EVI seasonality of these regions is mainly explained by the net loss or gain of leaves (Fig 2). In light-limited forests, minimum EVI was mostly not synchronized with maximum litterfall (Fig 2). This supports previous results showing that EVI browning is more likely related to leaf aging and parasites in these regions [26, 32, 61]. While in light-limited forests, leaf fall seasonality could be mainly explained by the coordination in time of leaf growth with senescence [8, 20, 23], in water-limited forests, leaf fall is not synchronous with leaf production (Fig 2). Leaf demography models [8] should account for this difference in order to be representative of the whole Amazon. More studies of leaf renewal based on field and remote remote sensing data [8, 20, 23] are needed to confirm the direct link between increase EVI and new leaves production.

## Conclusion

Subtropical and tropical seasons are generally defined in terms of dry or wet season, but here we show that this definition is not correct in the Amazonian forest regions without water constraints. In 70.4% of the Amazon forest, the increase in insolation triggers the visible progress of leaf growth and this process occurs in a sufficient proportion of the trees to be observed from satellites, just like during spring in temperate forests [62]. Direct and strong climate environmental signals trigger leaf growing season, which supports the hypothesis of a leaf production optimized for carbon gain under seasonal resource availability [56, 58, 60]. In absence of water limitation, the leaf production follows the solar insolation, while in water-limited regions, trees quickly produce leaves with the first rains to benefit from high insolation at the end of the dry season. This high dependency of seasonal leaf renewal on climate, previously largely underestimated [63], may indicate a high sensitivity of these ecosystems to climate change.

## Supporting information

S1 Fig Correlation between monthly maximal temperature from climate research unit [35] and monthly incoming solar radiation at the surface from CERES [36]. The data have been extracted from a grid of 0.5° spatial resolution over the studied area spanning from 40°W to 80°W in longitude and from 20°S to 10°N in latitude. The two variables have been normalized, using their monthly mean and standard deviation. The red dashed line is the identity line y = x and the Pearson’s correlation coefficient is given.
(EPS)

S2 Fig Seasonality of EVI, precipitation and maximal temperature described by the number of seasonal cycles during a year (12 indicates one peak every 12 months and 6 two peaks a year). (EPS)

S3 Fig Coefficient of determination of the model of EVI increase, from a linear model with only precipitation and maximal temperature (with a lag of 1 month for the whole area). The seasonal pattern of increasing EVI is predicted with an R^2^ above 0.6 for 85.1% of the region of interest. Regions shown are 1–Acre, 2–Roraima and 3–Venezuela. Detailed results are given in supplementary text for these regions.
(EPS)

S4 Fig Normalized monthly time series of EVI, precipitation, and maximal temperature for 10000 pixels in the regions Acre (region 1), Roraima (region 2) and Venezuela (region 3). (EPS)

S5 Fig Coefficient of determination (R^2^) of the model with only precipitation and maximal temperature (with a lag of 1 month for the whole area) and distribution of bamboo dominated forests in Acre extracted from the Brazilian vegetation map of 2005 [38]. (EPS)

S6 Fig Relation between elevations, MAIAC data characteristics and model fitting quality in the Roraima region. Elevation map of the Roraima region (region 2). Mean percentage of valid EVI MAIAC observations per months to estimate EVI and isoclines of altitude (b). Significative lag with maximal temperature (c). Quality of the fit (R^2^) of the model of EVI increase with precipitation and maximal temperature with a lag of 1 month and isoclines of altitude (d). Quality of the fit (R^2^) after accounting for the best time lag with maximal temperature and isoclines of altitude (e).
(EPS)

S7 Fig Estimated time lags in months and by pixels between the increase of EVI and the increase of maximal temperature. (EPS)

S8 Fig Quality of fit of the EVI model accounting for the time lags by pixels between the increase of EVI and the increase of maximal temperature, from a linear model with only precipitation and maximal temperature (time lag between 0 and 3 months). The seasonal pattern of increasing EVI is predicted with a R^2^ above 0.7 for 84.75% of the region of interest.
(EPS)

S9 Fig RMSE of the EVI model accounting for the time lags between the increase of EVI and the increase of maximal temperature (estimated by pixels), from a linear model with only precipitation and maximal temperature (with a time lag between 0 and 3 months). (EPS)

S10 Fig Association of annual climate variable statistics with climate controls. Quantile of mean annual precipitation (a), standard deviation of mean annual maximal temperature (b) and percentage of mean number of valid 8 day observations that were used to calculate the mean monthly values (c) in the five classes of climate associations with EVI. *Only pre* indicates pixels only associated with precipitation, *only tmx* indicates pixels only associated with maximal temperature, *pre* > *tmx* indicates that the part of EVI variance explained by precipitation is higher than the part of EVI variance explained by maximum temperature and *pre* < *tmx* indicates that the part of EVI variance explained by maximum temperature is higher than the part of EVI variance explained by precipitation. *No effect* indicates pixels where EVI had no associations with precipitation and maximal temperature.
(EPS)

S11 Fig Association of the the model fit (R^2^) with annual climate characteristics and number of EVI observations. Associations are presented for all the 1 km^2^ forested pixels in the studied area spanning from 40°W to 80°W in longitude and from 20°S to 10°N in latitude with annual precipitation (a), annual maximal temperature (b), percentage of mean number of valid 8 day observations that were used to calculate the mean monthly values (c), periods in the EVI signal (d), Altitude (e) and tree cover (f).
(EPS)

S1 Appendix Finding the months of the main EVI increase. (PDF)

S2 Appendix Periodicity analysis of monthly EVI MAIAC, precipitation and maximal temperature. (PDF)

S3 Appendix EVI MAIAC regional pattern analysis. (PDF)
