# Supplementary material for: Climate drivers of the Amazon forest greening
Source: PLoS One. 2017 Jul 14;12(7):e0180932. doi: 10.1371/journal.pone.0180932 (PMC5510836; doi:10.1371/journal.pone.0180932)
Supplement: S1 Appendix — (PDF) [file pone.0180932.s012.pdf]

S1 Appendix. Finding the months of the main EVI increase. To filter the EVI time series and find the main increase, we used a Fourier spectral decomposition. EVI inter-annual monthly mean values were modelled by a weighted sum of simple sine waves of period 6 months and 12 months, that is, one or two seasonal increases of EVI per year, as observed in the EVI time series. We assumed that other periods in the signal are only constituted by noise. To filter EVI, we fit the following equation to the EVI time series :

$$\widehat{EVI}(t) = EVI_0 + pow_0 \left(p_6 \sin(2\pi\frac{1}{6}t + \rho_6) + p_{12} \sin(2\pi\frac{1}{12}t + \rho_{12})\right) \quad (6)$$

with $p_6 + p_{12} = 1$ and for $t = 1, \ldots, 12 \times n$.

$\widehat{EVI}$ is the filtered time series, EVI_0 is as an estimate of the mean annual EVI, t is the time in month, ρ_6 and ρ_{12} are the delay of signal components with periods of 6 months and 12 months respectively. pow_0 is the power of the signal and p_6, p_{12} are the relative proportion of the periods of 6 months and 12 months respectively. The parameters EVI_0, pow_0, p_6, p_{12}, ρ_6 and ρ_{12} were estimated by least square minimization. To facilitate the optimization, the time series was replicated 3 times ($n = 3$).

After filtering the EVI signal, pits and peaks were identified in the $\widehat{EVI}$ time series. A peak or pit is an observation that is preceded and followed by, respectively, lower or higher observations [1, 2]. The main increase was defined as the months during the longest period between a pit and peak.

This analysis was also performed on precipitation and temperature time series to determine the relative proportion of the 6 and 12 months periods in their time series.

References

1. Kendall MG. Time-series, 2nd ed. Charles Griffin & Co, London; 1976.

2. Grosjean P, Ibanez F. pastecs: Package for Analysis of Space-Time Ecological Series; 2014. Available from: https://CRAN.R-project.org/package=pastecs.
