# Supplementary material for: Climate drivers of the Amazon forest greening
Source: PLoS One. 2017 Jul 14;12(7):e0180932. doi: 10.1371/journal.pone.0180932 (PMC5510836; doi:10.1371/journal.pone.0180932)
Supplement: S2 Appendix — (PDF) [file pone.0180932.s013.pdf]

S2 Appendix. Periodicity analysis of monthly EVI MAIAC, precipitation and maximal temperature. From the analysis of frequency in the time series of EVI and climate variables, we determined that 93.4% of the forested pixels have only one seasonal increase in the EVI signal per year (fig. S2 Figa). Some forested pixels, however, have two periods in the EVI signal and are spatially coherent. This could reflect an influence of the vegetation structure on EVI. Some forested pixels have two periods in the signal and are not clearly spatially aggregated, indicating large shifts in the signal. This might reflect noise in the data resulting from low availability of good quality observations for EVI computation. Regions where EVI signal shows more than one period in a year (6.6% of the forested surface) were localized largely in the Colombian Amazon, in the Brazilian States of Acre and Roraima, in the North-East part of Guyana, in the central and Eastern part Venezuela (fig. S2 Figa). Pixels with two periods in the EVI signal are not predictable if the number of periods in the climate variables are not correlated or the same. In the case of both climate variables with two periods (figs. S2 Figb-c) and EVI with only one period, our model won't fit the EVI data as it will predict two periods.
