# Supplementary material for: Climate drivers of the Amazon forest greening
Source: PLoS One. 2017 Jul 14;12(7):e0180932. doi: 10.1371/journal.pone.0180932 (PMC5510836; doi:10.1371/journal.pone.0180932)
Supplement: S3 Appendix — (PDF) [file pone.0180932.s014.pdf]

S3 Appendix. EVI MAIAC regional pattern analysis. In the following, we describe the EVI pattern for 3 regions (numbered in Fig. S3 Fig) where the model had problems to reproduce the seasonal pattern of EVI.

Brazilian region of Acre, region 1. In Acre, spatial aggregates of pixels show weak association to climate variables (R^2¡0.4, fig. S3 Fig region 1). The EVI signal in this region was found to have two distinct patterns (fig. S4 Fig region 1), a peak in November correlated with maximal temperature and a peak in June-July apparently dissociated of climate. The pixels with R^2 above 0.9 have one period per year while the pixels with R^2 below 0.1 have two periods per year (fig. S3 Fig region 1). The two periods are also visible in the analysis of EVI frequencies (fig. S2 Figa). Bamboo dominated forests are common in Acre and their occurrence have been mapped [1]. The patches with low R^2 appear spatially related to the bamboo dominated forests (fig. S5 Fig). These Bamboo dominated forests are characterized by a mosaic of forest stands without bamboo with heterogeneous forest structure, and stands of trees scattered within a canopy dominated by a single species of native arborescent bamboos (*Guadua spp.*) [2]. Our results show that MAIAC bidirectional reflectance distribution function (BRDF) may not describe the BRDF of such complex canopy structure accurately enough. Interestingly, the changes occurring in October and November indicate a good sensibility of EVI MAIAC to capture EVI changes during wet months (mean precipitation of 227.0 and 295.7 mm, respectively).

Brazilian region of Roraima, region 2. In Roraima (fig. S4 Fig region 2), spatially aggregated pixels showed only weak correlation to climate variables. The coefficient of determination (R^2) declined from over to 0.9 to below 0.1, from side to side of the hills (fig. S6 Figa). The areas with low R^2 values form plumes located on the West South-West sides of these hills (fig. S6 Figd). The MAIAC number of observations per month shows an increase from the top to the West and South-West side of these mountains (fig. S6 Figb) in the direction of the prevailing wind of this region. The highly significance of EVI increase in the areas with a R^2 above 0.9 is likely associated with maximal temperature increase occurring one month before. For the areas with a R^2 below 0.9, the general pattern of EVI is similar, however the increase occurs with a time lag of two or three months after the increase of maximal temperature. For these latter areas, the EVI increase was not correlated with precipitation, as EVI increase primarily followed maximal temperature. We estimated the lag of EVI increase with the increase of maximal temperature at 0, 1, 2 and 3 months. To discard artificial correlations between the climate variables, the lags were estimated only for the pixels where precipitation and maximal temperature are correlated below 0.2 during the increase of EVI at all the lags. Then, we compared the results of the model at lag 1 and the other lags using the Bayesian information criterion [3] and kept the best model (fig. S7 Fig). For this region, the time lags of two and three months are located on the West South-West sides of the mountains (fig. S6 Figc). The linear model of EVI with precipitation, maximal temperature and the associated lags allowed us to reproduce the pattern of EVI increase in Roraima (fig. S6 Fige). The lags are unlikely due to shade artifacts or slope effects because a BRDF function corrects for these effects in the MAIAC, and, if these artifacts were the causes, their effects should also occur in other regions of Amazonia. This region shows an unique climate seasonality in our data set, precipitation and maximal temperature are the most asynchronous and their peaks are separated by more than 5 months. In the areas where a time lag of two to three months was observed with respect to increase in solar radiation, trees may delay leaf flushing. The number of MAIAC observations per month is higher in these areas (fig. S6 Figb) meaning that they are less cloudy than the areas with a time lag of one month between EVI and maximal temperature increase. In the EVI signal, the increase of EVI for these area is less sharp (fig S4 Fig region 2).

Venezuela, Region 3. In Venezuela (fig. S3 Fig region 3 and fig. S4 Fig region 3), the observed EVI signal was found to be noisier than in other regions presented in this work. This region is amongst those with the lowest number of valid observations per month, a mean and maximum of 34.6 % and 50.7% was observed. Increase in EVI in associated to maximum temperature increase occurred from February and May and from July to October. This region has a complex climate with two periods in maximal temperature and precipitation per year.

References

1. Brazilian Institute of Geography and Statistics (IBGE) . Mapa de Vegetação do Brasil. Belem, Brazil.: IBGE; 2004.

2. Griscom BW, P Mark S Ashton. Bamboo control of forest succession: Guadua sarcocarpa in Southeastern Peru. Forest Ecology and Management. 2003;175(1-3):445–454. doi:10.1016/S0378-1127(02)00214-1.

3. Schwarz G. Estimating the dimension of a model. Annals of Statistics. 1978;6 (2):461–464.
